# A screen for transcription factor targets of Glycogen Synthase Kinase-3 highlights an inverse correlation of NFκB and Androgen Receptor Signaling in Prostate Cancer

**DOI:** 10.18632/oncotarget.2303

**Published:** 2014-08-07

**Authors:** Victor M. Campa, Eder Baltziskueta, Nora Bengoa-Vergniory, Irantzu Gorroño-Etxebarria, Radosław Wesołowski, Jonathan Waxman, Robert M. Kypta

**Affiliations:** ^1^ Cell Biology and Stem Cells Unit, CIC bioGUNE, Spain; ^2^ Department of Surgery and Cancer, Imperial College London, UK; ^3^ Present address: Instituto de Biomedicina y Biotecnología de Cantabria (IBBTEC), CSIC-Universidad de Cantabria, Santander 39011, Spain

**Keywords:** prostate cancer, glycogen synthase kinase-3, androgen receptor, NFκB transcription factor, Wnt signaling

## Abstract

Expression of Glycogen Synthase Kinase-3 (GSK-3) is elevated in prostate cancer and its inhibition reduces prostate cancer cell proliferation, in part by reducing androgen receptor (AR) signaling. However, GSK-3 inhibition can also activate signals that promote cell proliferation and survival, which may preclude the use of GSK-3 inhibitors in the clinic. To identify such signals in prostate cancer, we screened for changes in transcription factor target DNA binding activity in GSK-3-silenced cells. Among the alterations was a reduction in AR DNA target binding, as predicted from previous studies, and an increase in NFκB DNA target binding. Consistent with the latter, gene silencing of GSK-3 or inhibition using the GSK-3 inhibitor CHIR99021 increased basal NFκB transcriptional activity. Activation of NFκB was accompanied by an increase in the level of the NFκB family member RelB. Conversely, silencing RelB reduced activation of NFκB by CHIR99021. Furthermore, the reduction of prostate cancer cell proliferation by CHIR99021 was potentiated by inhibition of NFκB signaling using the IKK inhibitor PS1145. Finally, stratification of human prostate tumor gene expression data for GSK3 revealed an inverse correlation between NFκB-dependent and androgen-dependent gene expression, consistent with the results from the transcription factor target DNA binding screen. In addition, there was a correlation between expression of androgen-repressed NFκB target genes and reduced survival of patients with metastatic prostate cancer. These findings highlight an association between GSK-3/AR and NFκB signaling and its potential clinical importance in metastatic prostate cancer.

## INTRODUCTION

The protein kinase Glycogen synthase kinase-3 (GSK-3) is up-regulated in many cancers, including prostate cancer (PCa) [1–3] and GSK-3 inhibitors reduce prostate tumor growth *in vitro* [4, 5] and *in vivo* [6]. PCa initiation and progression are uniquely dependent on the androgen receptor (AR) [7]. AR transcriptional activity is regulated by co-activator and co-repressor proteins and by posttranslational modifications, such as phosphorylation by kinases [4, 8–11], including GSK-3, which is important for AR stability, nuclear localization and transcriptional activity [4, 5, 12]. The mechanism of action of GSK-3 is however complex and context-dependent, since its overexpression in some cell types inhibits AR [10, 11] and there are instances where GSK-3 inhibitors reduce proliferation of AR-negative PCa cells [13, 14]. In addition, GSK-3 regulates other signals, such as those mediated by Wnt and NFκB, which are themselves linked [15]. Adding further complexity, there are two GSK-3 isoforms, GSK-3α and GSK-3β that have different expression profiles in PCa [1]. Knockout studies have shown that the two isoforms generally compensate for one another [16]. However, there are examples where a single isoform has a unique or predominant function [17, 18]. This is the case in PCa, where GSK-3α is more important for maintaining AR transcriptional activity and silencing GSK-3β but not GSK-3α reduces PKB phosphorylation [1].

In order to identify isoform-specific GSK-3 targets in PCa, we screened for transcription factors whose binding to cognate DNA target sequences is altered upon GSK-3 silencing. We observed reduced binding to an AR target binding site upon silencing GSK-3α, consistent with our earlier study [1], and increased binding to an NFκB target-binding site in cells chronically silenced for GSK-3β. Further analyses using PCa cell lines and tumor array data revealed a link between GSK-3 expression and an inverse correlation between AR and NFκB signaling pathways. GSK-3 has previously been linked to NFκB activation [19] and there are a variety of reports on the mechanisms involved. For example, GSK-3 can inhibit p65 transcriptional activity [20], increase p105 stability [21], suppress chromatin accessibility [22] and inhibit IKK phosphorylation of IκB [23]. On the other hand, there are many instances where targeted deletion of GSK-3β inhibits NFκB activity [24–26]. The conclusion from these apparently conflicting studies is that GSK-3 regulation of NFκB is highly context-dependent and will only be understood by carrying out experiments using the cells and tissues of interest. The studies described in this report find that acute gene silencing and chemical inhibition of GSK-3 increase basal NFκB activity in PCa, and that combined inhibition of GSK-3 and NFκB signaling is more effective than inhibition of each alone for reducing PCa cell proliferation.

## RESULTS

### A screen for transcription factor targets of GSK-3 in PCa cells identifies unique and common targets of GSK-3α and GSK-3β

**Figure 1:**
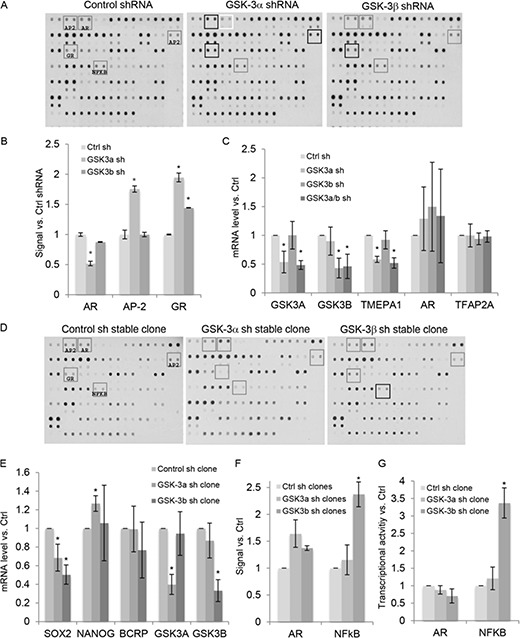
A screen for transcription factor targets of GSK-3 in PCa cells identifies AR and NFkB as targets of GSK-3α and GSK-3β, respectively **(A)** Scanned images of transcription factor DNA binding site arrays probed with nuclear extracts from 22Rv1 cells transiently transfected with the indicated shRNAs. The grey boxes highlight relevant transcription factor binding sites; changes in signal, compared with control shRNA-transfected cells, are indicated in black (increased binding to AP-2 and GR binding sites) and white (reduced binding to AR binding site). **(B)** Average signals for the indicated binding sites from two independent experiments in duplicate, relative to control shRNA; * p < 0.05, compared to control shRNA. **(C)** 22Rv1 cells transfected with indicated shRNA plasmids were selected with puromycin for 72 h and the expression of *GSK3A*, *GSK3B*, *TMEPA1, AR* and *TFAP2A* mRNAs determined by q-PCR; * p < 0.05 compared to control shRNA (Ctrl sh); n = 3. **(D)** Scanned images of representative transcription factor DNA binding site arrays probed with nuclear extracts from 22Rv1 cell clones expressing control, GSK-3α (clone 3) or GSK-3β (clone 18) shRNAs. Grey boxes highlight relevant transcription factor binding sites; black box shows increased signal at the NFkB binding site. **(E)** Analysis for the mRNA expression levels of *SOX2*, *NANOG*, *BCRP1*, *GSK3A* and *GSK3B* in 22Rv1 cell clones expressing control, GSK-3α (clone 3) or GSK-3β (clone 18) shRNAs was determined by q-PCR; * p < 0.05 compared to control shRNA (Ctrl sh); n = 3. **(F)** Average signals for the indicated binding sites for two independent cell clones for each in duplicate, relative to control cells; * p < 0.05, compared to control. **(G)** Gene reporter assays using extracts from 22Rv1 cell clones as in D. Basal NFκB activity was measured using NFκB-luciferase and AR activity was measured using MMTV-luciferase in cells cultured in charcoal-stripped medium with 10^−8^ M DHT. Renilla was used to normalize for transfection efficiency. Graphs show luciferase/renilla values, relative to control cells; * p < 0.05, compared to control; n = 2.

In order to identify isoform-specific GSK-3 targets in PCa, we screened for transcription factors whose binding to cognate DNA target sequences is altered upon GSK-3 silencing in 22Rv1 PCa cells. Since we previously found differences between the effects of acute and chronic silencing of GSK-3 on AR activity [1], experiments were carried out using both conditions. In the acute silencing experiments, we used nuclear extracts from cells transiently transfected with shRNAs specific for GSK-3α or GSK-3β. Acute GSK-3α silencing reduced the binding activity of AR to its DNA target element (Figures 1A and B), consistent with our previous study [1]. Also in keeping with these results, silencing of GSK-3α but not of GSK-3β reduced expression of the AR target gene TMEPA1 [27], but not of AR itself (Figure 1C). In addition, silencing GSK-3α increased binding activity of activator protein 2 (AP-2) (Figure 1A), without affecting expression of TFAP2A (Figure 1C), the major AP-2 family member expressed in 22Rv1 cells, and silencing GSK-3α and, to a lesser extent, GSK-3β increased binding activity of the glucocorticoid receptor (GR) (Figures 1A and B). We next carried out protein/DNA array screens using extracts from 22Rv1 cell clones stably silenced for either GSK-3α or GSK-3β. In contrast to the results from acute silencing, binding to AR target DNA was slightly increased in cells stably silenced for GSK-3α (Figures 1D and F). In addition, the changes in AP-2 and GR binding activities observed upon acute silencing of GSK-3α were not seen in stably silenced cells, suggesting that several changes induced upon acute silencing of GSK-3 are not maintained in stably silenced cell lines. Several other spots were reproducibly altered with stable GSK-3α silencing, namely an increase in binding to the Sp1 binding site (row 11 columns 1/2), the Stat5/6 binding site (columns 13/14) and the Pbx1 binding site (row 7 columns 15/16), also observed upon acute silencing of GSK-3α. To address the possibility that stable inhibition of GSK-3 leads to selection for prostate cancer stem/progenitor-like cells, as was recently reported for PC3 cells [28], we measured the expression levels of three genes expressed in 22Rv1-derived stem/progenitor cells, SOX2, NANOG and BCRP [29], in the 22Rv1-derived cell clones. This revealed small reductions in expression of SOX2 in both GSK- 3-isoform silenced cell lines, a small increase in NANOG expression in the GSK-3α-silenced cell line and no change in expression of BCRP (Figure 1E). These results suggest that stable inhibition of GSK-3 isoforms does not lead to selection for prostate cancer stem/progenitor-like cells. In addition, we observed increased binding to the NFκB target DNA sequence in cells stably silenced for GSK-3β (Figures 1D, F), and, consistent with this, NFκB-dependent transcription was increased in cell lines stably silenced for GSK-3β (Figure 1G).

### GSK-3 inhibition activates NFκB independently of β-catenin and AR

**Figure 2:**
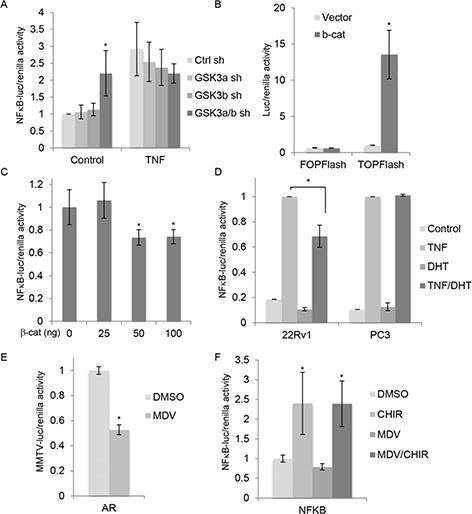
GSK-3 inhibition activates NFκB independently of β-catenin and AR **(A)** Gene reporter assays using extracts from 22Rv1 cells transfected with control shRNA (Ctrl sh) or shRNAs targeting GSK-3α (GSK3a sh), GSK-3β (GSK3b sh) or both GSK3a/b sh), and plasmids encoding NFκB-luciferase and renilla (pRL-tk), and treated with TNF (20 nM) or vehicle for 6 h. Graph shows luciferase/renilla ratios 18 h after transfection normalized to Ctrl sh; * p < 0.05 vs. Ctrl sh; n = 4. **(B)** Gene reporter assays using extracts from 22Rv1 cells transfected with plasmid encoding β-catenin or empty vector, Super8xTOPFlash or Super8xFOPFlash and renilla. Graph shows luciferase/renilla ratios normalized to vector; * p < 0.01 vs. vector. **(C)** Gene reporter assays using extracts from 22Rv1 cells transfected with the indicated amounts of plasmid encoding β-catenin (total amount brought to 200 ng with empty vector), and plasmids encoding NFκB-luciferase and renilla. Graph shows luciferase/renilla ratios, normalized to 0 ng β-catenin plasmid; * p < 0.05 vs. vector. **(D)** Gene reporter assays using extracts from 22Rv1 cells and PC3 cells transfected with NFκB-Luc and renilla. After transfection, cells were cultured for 18 h in phenol red-free medium containing charcoal-stripped serum with or without 10 nM DHT and then treated with 20 nM TNF for 6 h before measuring luciferase activities. Graph shows luciferase/renilla ratios; TNF-induced activity is set to 100% in each cell line; * p < 0.05; n = 3 (22Rv1), n = 2 (PC3). **(E)** Gene reporter assays using extracts from 22Rv1 cells transfected with MMTV-luc and renilla, treated with carrier (DMSO) or the AR antagonist 10 uM MDV3100 (MDV) and AR activity determined as in Figure 1F; *p < 0.05 vs. control; n = 2. **(F)** Gene reporter assays using extracts from 22Rv1 cells transfected with plasmids encoding NFκB-luciferase and renilla and treated with carrier (DMSO), 5 uM CHIR99021 (CHIR) and 10 uM MDV3100 (MDV), as indicated. Graph shows luciferase/renilla ratios normalized to DMSO; * p < 0.05 vs. DMSO; n = 2.

The observation that stable but not acute silencing of GSK-3β increased NFκB activity prompted us to determine the effects of acute silencing on NFκB-dependent transcription. Acute silencing of single GSK-3 isoforms did not affect NFκB transcriptional activity (Figure 2A), consistent with no change in NFκB target DNA binding being observed (Figure 1A and data not shown). However, silencing of both GSK-3 isoforms together modestly but significantly increased basal NFκB transcriptional activity (Figure 2A). In addition, NFκB transcriptional activity was increased by treatment of cells with the specific GSK-3 inhibitor CHIR99021 [30] (see Figure 2F). In summary, chronic silencing of GSK-3β and acute inhibition of GSK-3α/β led to activation of NFκB, as detected by increased DNA target binding and transcriptional activity. We therefore decided to explore the effects of GSK-3 inhibition on NFκB signaling in more detail.

Since silencing of both GSK-3 isoforms together also activates Wnt/β-catenin signaling in 22Rv1 cells [1], we wished to determine if stabilization of β-catenin was responsible for the observed activation of NFκB. Cells were transfected with a plasmid encoding stabilized β-catenin, which increased β-catenin/Tcf-dependent transcription as expected (Figure 2B). β-catenin inhibited NFκB-dependent transcription (Figure 2C), suggesting that the effect of GSK-3 inhibition on NFκB is not mediated by β-catenin. In addition to increasing basal NFκB activity, GSK-3 inhibitors reduce AR-dependent transcription [4]. Given previous reports that androgens inhibit NFκB [31, 32], we hypothesized that GSK-3 inhibition leads to NFκB activation by inhibiting AR. To test this possibility, we first examined the effect of AR activation on NFκB activation by Tumor Necrosis Factor (TNF). Cells were stimulated with TNF in the presence or absence of the AR ligand DHT. DHT treatment reduced TNF activation of NFκB in 22Rv1 cells (Figure 2D) and, to a lesser extent, in LNCaP cells (data not shown), but not in PC3 cells, which do not express AR (Figure 2D). These results suggest that AR could play a role in the activation of NFκB upon GSK-3 inhibition. To determine the impact of AR on GSK-3 inhibitor-mediated activation of NFκB, gene reporter assays were carried out in the presence of the AR antagonist MDV3100. As expected, MDV3100 inhibited AR transcriptional activity (Figure 2E). However, it did not affect NFκB activation by CHIR99021 (Figure 2F), suggesting that the effects of GSK-3 on NFκB are not mediated via AR.

### Inhibition of GSK-3 activates NFκB via RelB

**Figure 3:**
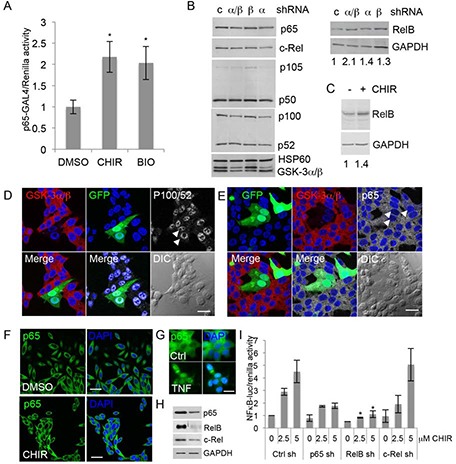
Inhibition of GSK-3 activates NFκB via RelB **(A)** Gene reporter assays using extracts from 22Rv1 cells transfected with plasmids encoding p65-Gal4 Gal4-Luc, renilla and treated with vehicle (DMSO) or 5 uM CHIR99021 (CHIR) or 2.5 uM BIO-Acetoxime (BIO). Graph shows luciferase/renilla ratios normalized to DMSO; * p < 0.05 vs. DMSO; n = 2. **(B)** Western blots of extracts from 22Rv1 cells transfected with control shRNA (c) or shRNAs targeting GSK-3α (α), GSK-3β (β) or both isoforms (α/β), selected with puromycin for 72 h and probed for the indicated NFκB family proteins and HSP60 and GAPDH as loading controls. Numbers below the GAPDH blot indicate relative RelB signal normalized to GAPDH. Densitometry analysis (ImageJ) from 3 experiments indicated that silencing both GSK-3 isoforms together increased RelB 1.6 to 2.1-fold, compared to control shRNA (p < 0.05). **(C)** Western blots of extracts from 22Rv1 cells treated with 2.5 uM CHIR99021 (CHIR) or vehicle (DMSO) for 16 h were probed for RelB and GAPDH as a loading control. Densitometry analysis from 3 experiments indicated that RelB was 1.4 to 2.3-fold higher in CHIR99021-treated cells than in DMSO-treated cells (p < 0.05). (D, E) Immunostaining for GSK-3α and GSK-3β (red) and p100/p52 **(D)** or p65 **(E)** (white) in 22Rv1 cells transfected with shRNAs to silence both GSK-3 isoforms and GFP plasmid (green) to visualize transfected cells. DAPI staining of nuclei is shown in blue. Arrows indicate cells silenced for GSK-3; scale bars 25 μm. **(F)** Immunostaining for p65 (green) in 22Rv1 cells treated with vehicle (DMSO) or CHIR99021 (CHIR) for 16 h. DAPI staining of nuclei is shown in blue; scale bar 25 μm. **(G)** Immunostaining for p65 (green) in 22Rv1 cells with vehicle (Ctrl) or TNF for 3 h. DAPI staining of nuclei is shown in blue; scale bar 62.5 μm. **(H)** Western blots of extracts from 22Rv1 cells transfected with control (left) or the indicated shRNA plasmid (right), selected with puromycin for 72 h and probed for p65, RelB and c-Rel; GAPDH was used as a loading control (blot shown is for RelB shRNA). **(I)** Gene reporter assays using extracts from 22Rv1 cells transfected with control (Ctrl), p65, RelB and c-Rel shRNAs, selected with puromycin for 72 h, then transfected with NFκB-luciferase and renilla and treated with DMSO or CHIR99021 at 2.5 uM and 5 uM. Graph shows luciferase/renilla ratios normalized to DMSO in Ctrl cells; * p < 0.05 vs. Ctrl sh at the same dose of CHIR99021; n = 2.

NFκB consists of a family of transcription factors whose subunits p65 (RelA), RelB, c-Rel, and p50 and p52, generated from the precursors p105 and p100, respectively, form dimers that regulate gene expression. IκB proteins sequester NFκB dimers in the cytoplasm and activation of IκB kinase (IKK) results in IκB degradation and the release of NFκB [33]. To determine if NFκB activation upon GSK-3 inhibition involves IκB, we used the Gal4-p65/Gal4-luc system [34], in which a fusion protein formed by the Gal4 DNA-binding domain and the p65 transactivation domain drives expression of Gal4-responsive luciferase independently of IκB. Treatment of 22Rv1 cells with CHIR99021 or the unrelated GSK-3 inhibitor BIO-Acetoxime (BIO) increased p65-Gal4 activity (Figure 3A), suggesting that GSK-3 inhibition activates NFκB signaling independently of IκB.

To determine if the effects of GSK-3 inhibition involved changes in NFκB family proteins, we next examined their expression levels by western blotting. Silencing of both GSK-3 isoforms together did not significantly affect most NFκB family members, although there was a trend for a reduction in the p100/p52 ratio. However, silencing GSK-3 did result in a small but significant increase in RelB (Figure 3B). In support of the gene silencing results, CHIR99021 also increased RelB levels (Figure 3C). Since GSK-3 regulates the subcellular localization of many proteins, we examined the effects of GSK-3 silencing on the localization of p100/52 and p65 (RelB could not be readily detected using the available antibodies). P100/52 was found mostly in the nucleus (Figure 3D) and p65 mostly in the cytoplasm (Figure 3E), and neither one was affected by GSK-3 silencing. Changes in p65 were also not detected in cells treated with CHIR99021 (Figure 3F). In contrast, treatment of 22Rv1 cells with TNF induced nuclear translocation of p65 (Figure 3G). Together, these results raise the possibility that activation of NFκB upon inhibition of GSK-3 results from an increase in RelB. To investigate the role of RelB, gene reporter assays were carried out in 22Rv1 cells silenced for p65, RelB and c-Rel [35] (Figure 3H). Silencing RelB and, to a lesser extent p65, but not c-Rel, reduced CHIR99021-mediated activation of NFκB (Figure 3I), indicating that RelB plays a predominant role in the response to CHIR99021.

### Inhibition of NFκB potentiates the inhibitory effect of CHIR99021 on PCa cell proliferation

**Figure 4:**
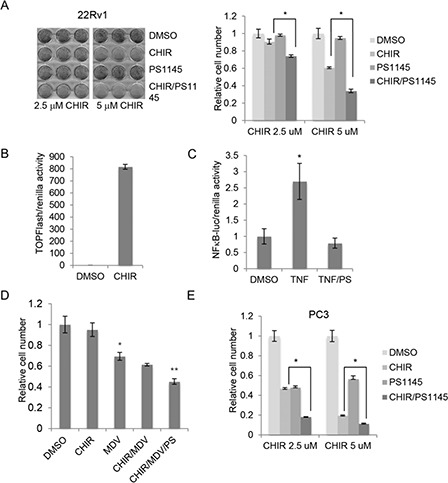
Inhibition of NFκB potentiates the inhibitory effect of CHIR99021 on PCa cell proliferation **(A)** Left: representative experiment showing crystal violet staining of 22Rv1 cells cultured for 1 week with DMSO, 10 uM PS1145 and/or the indicated concentrations of CHIR99021 (CHIR). Right: absorbance (540 nm) of solubilized crystal violet was used to calculate relative cell number; *p < 0.01. **(B)** β-catenin/Tcf-dependent transcriptional activity in 22Rv1 cells treated with carrier (DMSO) or 5 μM CHIR99021 (CHIR) was determined by gene reporter assay using Super8xTOPFlash normalized to renilla. **(C)** NFκB-dependent transcriptional activity in 22Rv1 cells treated with carrier or TNF and 10 uM PS1145 (PS) as determined by gene reporter assay using NFκB-luciferase normalized to renilla; *p < 0.01 vs. DMSO. **(D)** Quantitation of crystal violet assays showing relative cell number for 22Rv1 cells cultured for 1 week in the presence of carrier (DMSO) −/+ 1 uM CHIR99021 (CHIR), 10 uM MDV3310 (MDV) and 10 uM PS1145 (PS); p < 0.05 versus DMSO (*) and versus MDV (**). **(E)** Quantitation of crystal violet assays showing relative cell number for PC3 cells cultured for 1 week in the presence of DMSO, 10 uM PS1145 and/or the indicated concentrations of CHIR99021 (CHIR); *p < 0.01.

Since activation of NFκB promotes cancer cell survival, we hypothesized that combined inhibition of GSK-3 and NFκB would inhibit proliferation in PCa cells. To test this possibility, we treated 22Rv1 cells with CHIR99021 to inhibit GSK-3 and PS1145 to inhibit NFκB signaling. As expected, given the low basal NFκB activity in 22Rv1 cells, PS1145 alone had no significant effect on cell number (Figure 4A). However, PS1145 potentiated the effects of CHIR99021, consistent with our hypothesis. The activities of the inhibitors were confirmed using gene reporter assays: CHIR99021 increased β-catenin activity (Figure 4B) and PS1145 inhibited TNF-induced NFκB activation (Figure 4C). Since GSK-3 inhibition represses AR signaling, we also tested the effects of combined inhibition of GSK-3, NFκB and AR on cell proliferation using MDV3100. In these experiments we used a dose of CHIR99021 that had no significant effect on proliferation when used alone (Figure 4D). As expected, MDV3100 reduced 22Rv1 cell proliferation and this effect was slightly enhanced by CHIR99021. Importantly, combined treatment of cells with MDV3100, CHIR99021 and PS1145 further reduced cell number (Figure 4D). We also examined the effects of combined inhibition of GSK-3 and NFκB in PC3 cells, which do not express AR and have constitutively high NFκB activity. Single treatments with PS1145 and CHIR99021 significantly reduced PC3 cell number, and the combination of both inhibitors had an additive effect (Figure 4E). These results suggest that combined inhibition of GSK-3 and NFκB may also be an effective for androgen-independent PCa.

### GSK3 expression is associated with an inverse correlation between AR and NFκB signaling in prostate tumors

**Figure 5:**
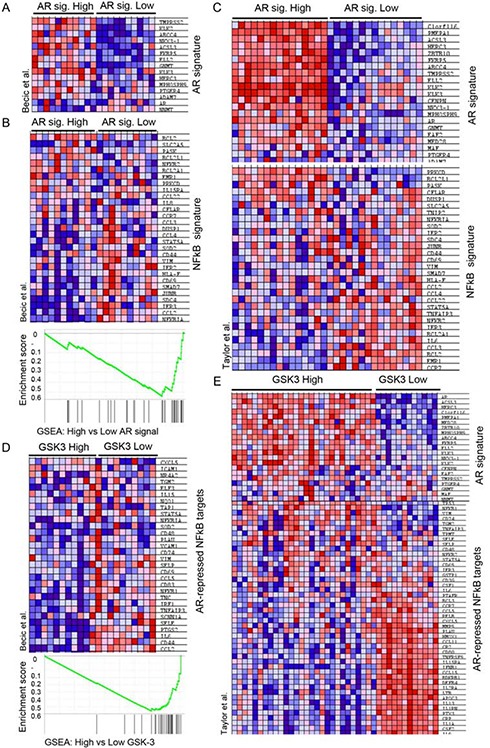
GSK3 expression is associated with an inverse correlation between AR and NFκB signaling in prostate tumors **(A)** Heatmap showing the expression of AR target genes in tumors with elevated expression (highest summed Z-score) versus tumors with reduced expression (lowest summed Z-score) of the indicated genes; dataset: Becic-00235 in the CaArray database. **(B–E)** Gene Set Enrichment Analysis (GSEA) of NFκB target genes in human tumors **(B, C)** Heatmap and enrichment score of the NFκB gene signature [39] in tumors with elevated expression of AR target genes (highest summed Z-score; high AR signature) versus tumors with reduced expression (lowest summed Z-score; low AR signature); datasets Becic-00235 (B) and GSE21034 (GEO) (C); p < 0.01. **(D, E)** Heatmap and enrichment score of NFκB-target genes with reduced expression in tumors with elevated androgen signaling (AR-repressed NFκB-targets) in tumors with elevated expression (Z-score > 0.5; GSK-3 High) versus reduced expression (z-score < 0.5; GSK-3 Low) of both GSK-3 isoforms; datasets Becic-00235 (D) and GSE21034 (GEO) (E); p < 0.01.

**Figure 6:**
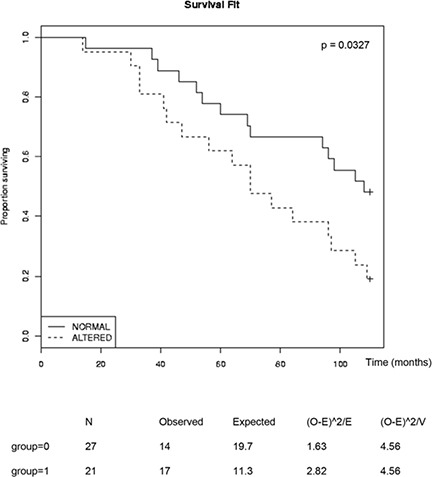
Inverse correlation of survival of patients with metastatic prostate cancer and expression of AR-repressed NFκB-targets Kaplan Meier plots were obtained from the cBio Cancer Genomics portal, analyzing the Michigan metastatic prostate adenocarcinoma dataset (ref [41]) and the AR-repressed NFκB-targets from Figure 5 (*CSF1, IL6, PTAFR, BCL3, CCR7, CCL5, RELB, CXCL5, MMP9, PLAU, HMOX1, CCL11, CR2, CD80, TNFRSF9, IL15RA, IFNB1, CCL15, BDKRB1, IL2RA, LTB, APOC3, IL13, IL1RN, PTX3, CRP, IL1A, CSF2* and *IL9*). Statistical significance of the whole sample was determined with the Log Rank test at 100 months p= 0.0327; Chi-Square test = 4.6 on 1 degree of freedom.

The observed effects of combined inhibition of GSK-3, NFκB and AR prompted us to look for possible links among these signaling pathways in human tumors. To this end, we carried out gene set enrichment analysis (GSEA) [36] of human tumors from a previously published database [37]. Tumors were first sorted based on the summed Z-score of the expression levels of a set of genes that is regulated by androgen signaling (AR signature) [38]. Tumors with the highest summed Z-scores were classified as “high AR signature” tumors and those with the lowest values were classified “low AR signature” tumors (Figure 5A). GSEA was then used to examine NFκB pathway activity within these two groups using an NFκB target gene signature [39]. The NFκB target gene signature was significantly diminished in tumors with the high AR signature, supporting the hypothesis that AR signaling inversely correlates with NFκB signaling (Figure 5B). This was also observed in an independent tumor dataset [40] (Figure 5C). We next examined NFκB target gene expression levels in tumors with high or low expression of *GSK3A* and *GSK3B*. Tumors with a positive Z-score (compared to normal prostates) of both GSK-3 isoform mRNAs were classified as “high GSK-3”, and tumors with a negative score for both isoforms were classified as “low GSK-3”. Although this classification only reflects GSK-3 mRNA expression (above or below that in normal prostate) and not kinase activity, it revealed that tumors with low GSK-3 expression were enriched for androgen-repressed NFκB target genes (Figures 5D, E). Finally, in order to determine whether expression of the androgen-repressed NFκB target genes identified has prognostic potential in prostate cancer, we analyzed a publicly available patient data set with follow-up data from the cBio Cancer Genomics portal [41]. This revealed that high expression of androgen-repressed NFκB target genes is significantly correlated with reduced survival in patients with metastatic prostate cancer (Figure 6). Together, these findings highlight an inverse correlation between GSK-3/AR and NFκB signaling in patient tumors with potential clinical importance in metastatic prostate cancer.

## DISCUSSION

The involvement of GSK-3 in cancer is complex, with evidence supporting roles for this kinase as a tumor suppressor and a tumor promoter. Further complexity arises from the existence of multiple GSK-3 isoforms with differences in expression, subcellular localization and substrate preference. GSK-3 inhibitors are currently in clinical trials for the treatment of metastatic pancreatic cancer [42], and have the potential to be used to treat several other types of cancer, including PCa. Given the complex responses to GSK-3 in different cell types, it will be important to identify tumor-specific GSK-3 targets as biomarkers for effective treatment. In addition, it is essential to identify targets that may have undesired effects on tumor cell survival or proliferation. The present study reveals that inhibition of GSK-3 in PCa cells leads to activation of NFκB, and that this reduces the impact of GSK-3 inhibition on cell proliferation. These observations suggest that combined inhibition of GSK-3 and NFκB may be required for effective inhibition of prostate tumor growth.

In this report we have focused on NFkB and its relationship with AR. However, our analysis of GSK-3 targets revealed additional potential targets and, in addition, differences between stably silenced and transiently silenced cells that will require further analysis. We noted in our earlier study [1] that while there were no significant differences in growth rates of the stable cell lines in short-term (72 h) proliferation assays, cell lines with reduced levels of either GSK-3α or GSK-3β formed fewer colonies than control cells in colony formation assays. Moreover, when colony formation assays were conducted in hormone-depleted medium, GSK-3α-silenced cell lines formed similar numbers of colonies as control cells, whereas GSK-3β-silenced cells formed fewer colonies. Those results suggested that chronic silencing of GSK-3α has an impact on hormone-dependent growth, while chronic silencing of GSK-3β affects both hormone-dependent and hormone-independent growth [1]. However, the mechanisms that underlie the different transcriptional consequences of short-term versus long-term GSK-3 inhibition are not known. One possibility is that stable inhibition of GSK-3 isoforms leads to selection for different cell subsets or types, or changes the proportions of precursor cells in the population. Consistent with this possibility, small molecule inhibition of GSK-3 was recently reported to deplete the population of prostate cancer stem/progenitor-like cells in PC3 and PC3M cells, as measured by the proportion of ALDH^HIGH^ cells and reduced expression of stem/progenitor markers including NANOG and OCT4 [28]. However, this was not observed in LNCaP C4-2 and DU145 cells. Our analysis of 22Rv1 stem/progenitor cell marker gene expression revealed a small increase in NANOG and reduction in SOX2 in GSK-3α-silenced cells (Figure 1E), inconsistent with selection for prostate cancer stem/progenitor-like cells. Nevertheless, it is interesting to note that treatment of PC3M cells with a small molecule inhibitor of GSK-3 also had opposite effects on expression of NANOG and SOX2. Further studies will be required to determine if changes such as these reflect a change in the proportion of a specific population of progenitor cells.

As outlined in the Introduction, GSK-3 has previously been linked to NFκB activation and the mechanisms involved may be highly context-dependent. Our studies found that acute gene silencing and chemical inhibition of GSK-3 increase basal NFκB activity. Inhibition of GSK-3 in serum-starved cells has previously been reported to activate NFκB via degradation of IκB and nuclear translocation of p65 [19, 43]. This does not appear to be the mechanism in PCa cells, since p65 remained in the cytoplasm in CHIR99021-treated cells (Figure 3F) and inhibition of GSK-3 increased p65-Gal4 activity (Figure 3A), which suggests activation takes place downstream of IKK/IκB. Instead, we observed increased levels of RelB upon GSK-3 inhibition, as has been reported in Jurkat cells, where GSK-3 was found to promote RelB degradation [44]. The importance of RelB in PCa is underlined by its increased expression in high Gleason score tumors [45], and by a study in which gene silencing of RelB reduced PCa cell tumorigenicity [46]. Although we did not detect changes in p65 levels or nuclear localization upon GSK-3 inhibition, the results of the p65-Gal4 reporter assays (Figure 3A) and the gene silencing experiments (Figure 3I) suggest that p65 may play an indirect role in GSK-3 inhibitor-induced NFκB activation. Such a role could result from the known cross-regulation of p65 and RelB signaling, which takes place at several levels [33].

Analysis of AR and NFκB transcriptional activities in PCa cell lines suggests an inverse association that also correlates with GSK-3. One possibility is that a reduction in GSK-3 signaling activity, for example resulting from loss of PTEN, leads to activation of NFκB, which then inhibits AR signaling. This would be inconsistent with the view that NFκB increases AR expression and/or activity [47–49], although there is also evidence that NFκB inhibits AR in androgen-dependent PCa [50]. A second possibility is that inhibition of GSK-3 reduces AR activity, which then leads to NFκB activation. This would be consistent with our observation and other reports that activation of AR inhibits NFκB [31, 32]. However, the AR antagonist MDV3100 did not increase basal NFκB activity or potentiate GSK-3 inhibitor-mediated NFκB activation (Figure 2F), suggesting that this not the case in 22Rv1 cells. A third possibility is that GSK-3 independently regulates AR and NFκB and their relative activities are determined by the activation state of GSK-3. This possibility would be consistent with the gene expression data, which showed an inverse correlation between androgen- and NFκB-dependent target gene expression in PCa that correlates with GSK-3 mRNA expression. In addition, we observed a correlation of ‘androgen-repressed NFκB target’ gene expression and reduced survival of patients with metastatic prostate cancer (Figure 6). While this manuscript was in review, a study was published of the effects of increased NFκB activity and androgen-depletion on gene expression in non-tumorigenic mouse prostates. This led to the identification of a gene expression signature that predicted disease-specific survival and distant metastases-free survival in patients with PCa [51]. Intriguingly, the genes in that signature do not show any overlap with the genes we analyzed here. Further studies will be required to establish if these tumor signatures arise from specific misregulation of NFκB- and androgen-dependent signaling in a subset of tumor cells, or from tumors of different cellular compositions.

Our results suggest that combining GSK-3, AR and NFκB inhibitors will provide a more effective therapy than single agents for the treatment of some prostate tumors. A combination of GSK-3 and NFκB inhibitors was recently proposed for treatment of osteosarcoma [52]. In this example, however, inhibition of GSK-3 reduced NFκB activity, once again underlining the context-dependent nature of the link between GSK-3 and NFκB signaling. Importantly, since silencing GSK-3α alone reduces AR activity without activating NFκB, our results support the case for the development and sequential use of isoform-specific GSK-3 inhibitors [53] to treat different stages of PCa.

## METHODS

### Cell culture and proliferation assays

22Rv1 cells (ATCC) were cultured in RPMI 1640/DMEM (1:1) (Invitrogen) supplemented with 20% FCS and antibiotics (100 U/ml penicillin, 100 μg/ml streptomycin) at 37°C and 5% CO_2_. LNCaP and PC3 cells (ATCC) were cultured in RPMI 1640 supplemented with 10% FCS and antibiotics. Cells were passaged when they reached 70–80% confluence at 1:5–6 using 0.05% trypsin. For proliferation assays, cells were seeded at a density of 1,000 cells/cm^2^ in triplicate in 12-well plates and inhibitors or an equal volume of carrier were added the next day. Media were replaced and fresh inhibitors added every other day for 7 days. Cells were then rinsed with PBS, stained with crystal violet, rinsed again, air-dried and images acquired. In some experiments, crystal violet was subsequently solubilized in 10% acetic acid and absorbance measured at 590 nm. The GSK-3 inhibitors CHIR99021 (Axon Medchem and Calbiochem) and BIO-acetoxime (Merck), the IKK inhibitor PS1145 (Sigma) and the AR inhibitor MDV3100 (Selleck Chemicals LLC) were all dissolved in DMSO. TNF (R&D) was dissolved in PBS + 0.1% BSA and DHT (Sigma) in ethanol.

### RNA analysis

Reverse transcription was performed on 1 ug of total RNA using M-MLV Reverse Transcriptase and RNase OUT Ribonuclease Inhibitor (Invitrogen), according to manufacturer's instructions. Quantitative-PCR was performed using PerfeCTa Sybr Green Supermix, Low Rox (Quanta, Barcelona, Spain) in a Viia7 Real-Time PCR System (Applied Biosystems). The primer sequences were previously described [54–56], apart from GSK3A AGGTCCCCAGCGGGCACTAC, GGGTAGGTGTGGCATCGGTCG; GSK3B GTCCTGGGAACTCCAACAAGGG, GTGAAATGTCCTGTTCCTGAC; and TFAP2A ACGTTACCCTGCTCACATCAC, CAGGAAATTCGGTTTCGCACA. Relative fold changes in mRNA were determined according to the ΔΔCt method, relative to the housekeeping gene 36B4, and intraexperimental standard deviation (s.d.) was calculated according to Bookout and Mangelsdorf [57]. Statistical significance was calculated from three independent experiments using Student's t test.

### Plasmids and transfections

Plasmids for β-catenin (pmT23 β-catenin) [58], pSM2c GSK-3α (αsh1 and αsh2), GSK-3β (βsh1 and βsh2) and control shRNA plasmid (Open Biosystems, Madrid, Spain) [59, 60], RelA, RelB and c-Rel shRNAs [35] (from B. Lewis (NCI, Bethesda, MA, USA) and pSUPER-Retro-puro control shRNA [61] (from A. Carracedo, CIC bioGUNE) were previously described. Gene reporter assays were carried out using NFκB-luciferase (Clontech), pGal4, pGal4-p65 [62] and Gal4-luciferase (from the BCCM/LMBP, Ghent University/Lienhard Schmitz, Institute of Biochemistry, University of Giessen), pRL-TK (Promega), MMTV-luciferase (from C. Bevan, Imperial College London) and Super8xTOP/FOPFlash [63] (from R. Moon, University of Washington, Seattle). Cells were plated at a density of 50,000 cells/cm^2^ (22Rv1) or 25,000 cells/cm^2^ in 24-well plates and 24–48 hours later transfected using 1 μl of Lipofectamine, 2 μl of Plus reagent, 100 ng of firefly luciferase reporter, 0.5 ng pGal4/pGal4-p65 (where indicated), 15 ng of pRL-TK and up to 200 ng (unless otherwise indicated) of shRNA or expression plasmids, according to manufacturer's instructions (Invitrogen). Luciferase activity was measured with the DualGlo Stop&Glo luciferase assay system (Promega). For experiments using MMTV-luciferase, cells were cultured in phenol red-free RPMI and 10% charcoal-stripped serum.

### Transcription factor binding screen

Cells were rinsed in PBS and nuclear extracts were prepared using the Nuclear Extraction Kit (Panomics). 5 μg of nuclear extract was used for hybridization with the TranSignal™ Protein/DNA Array I (Panomics), according to manufacturer's instructions. In brief, nuclear extracts were incubated with a pool of biotin-labeled transcription factor (TF) binding probes, and DNA/TF complexes were isolated using an ion-exchange column. Finally, the isolated DNA probes were hybridized to membranes containing complementary TF binding site sequences and bound probes were visualized using an HRP-conjugated anti-biotin antibody. Quantitation was done by selecting non-saturated exposures of high-resolution scans of the membranes, grid circles and calculating the average signal using ImageJ software, using negative controls as a blank and taking the average signal from the row of positive controls for each membrane to normalize the data for each membrane. Binding assay experiments were carried out at least twice each, and, in the case of the stable lines, using two different shRNAs for each GSK-3 isoform.

### Western blotting

Cells were rinsed in PBS and lysed in RIPA (radioimmunoprecipitation assay) lysis buffer (19) containing Complete™ Protease Inhibitor Cocktail (Roche) and PhosSTOP Phosphatase Inhibitor Cocktail (Roche) on ice for 15 minutes, cleared by centrifugation and used directly or frozen on dry ice for later use. 20 μg of lysate was resolved on 8% or 10% SDS polyacrylamide gels, transferred to nitrocellulose membranes (Whatman). Nonspecific binding was blocked using 3% BSA/TBST (TBS with 0.05% Tween-20), and blots were probed overnight at 4°C with primary antibodies. Blots were washed in TBST and antigens were detected using HRP-conjugated secondary antibodies as previously described [1, 59]. Primary antibodies, diluted 1:1000, were to GSK-3α (Santa Cruz Biotechnology sc-5264), GSK-3β (BD Biosciences 610202), HSP60 (Santa Cruz Biotechnology), GAPDH (Sigma), and to NFkB p65 (D14E12), RelB (C1E4), c-Rel, p105/p50 and p100/p52 (18D10) (all Cell Signaling).

### Immunofluorescence

Cells were plated on gelatin-coated glass coverslips 48 h before transfection. Cells were fixed for 15 min in 4% PFA, permeabilized with 0.1% Triton for 10 min, incubated for 1 h with blocking buffer (50 mM glycine, 2% BSA, 0.01% Na-azide in PBS) to block nonspecific binding and probed for 2 h with primary antibodies diluted in wash buffer (blocking buffer 1:10 in PBS). Primary antibodies used were to GSK-3α (1:500, Santa Cruz Biotechnology sc-5264), GSK-3β (1:500, BD Biosciences 610202), p65 (1:200, Santa Cruz Biotechnology) and p100/52 (1:250, Abcam). Immune complexes were detected with appropriate Alexa488- or Alexa594-congugated secondary antibodies (Invitrogen) diluted 1:500 in wash buffer and mounted using Vectashield^®^ (Vector Labs), and DAPI (diamidino-2-phenylindole) was used to stain nuclei. Confocal images were acquired as previously described [59] with an SP2 microscope station (Leica) using a 63 × 1.3 NA lens.

### Analysis of microarray data

The human prostate cancer gene expression datasets used (Becic [37]; Taylor [40]) are available at caArray (https://array.nci.nih.gov/caarray/home.action) and cBio Cancer Genomics (www.cbioportal.org/public-portal/), respectively. Cell files containing raw data were normalized with RMAExpress (Robust Multichip Average) software, and chips with normalized unscaled standard error (NUSE) or relative log expression (RLE) values 1.5 IQR (interquartile range) above the upper quartile or 1.5 IQR below the lower quartile were discarded in further analysis. The intensity of the androgen-responsive-gene signature [38] was then scored using the Z-score values calculated for the genes included in the signature, and those tumors with highest or lowest added Z-score were classified as high or low AR-signature, respectively. Similarly, the Z-score was calculated for GSK3A and GSK3B and tumors with a Z-score value of GSK3A and GSK3B > 0.5 were classified high total GSK3, and tumors with a Z-value < −0.5 for both isoforms were classified as low total GSK3. Gene set enrichment analysis (GSEA) [36] was performed with the normalized gene expression values from the tumors classified as above (high vs. low AR signature; high vs. low GSK3), and the level of enrichment of the NFκB signature [39] was calculated using Student's t test on the collapsed probe sets and running 1000 permutations. Kaplan Meier plots were obtained from cBio Cancer Genomics portal analyzing the Michigan metastatic prostate adenocarcinoma dataset [41]. Statistical significance of the whole sample was determined with the log rank test both at 100 months and 220 months, with the former found to be significant.

## Abbreviations

GSK-3: (glycogen synthase kinase-3)
AR: (androgen receptor)
GR: (glucocorticoid receptor)
CHIR: (CHIR99021)
BIO: (BIO-Acetoxime)
PS: (PS1145)
NFκB: (nuclear factor kappa-light-chain-enhancer of activated B cells)
PCa: (prostate cancer)
DHT: (dihydrotestosterone)
TNF: (tumor necrosis factor)
AP-2: (activating protein 2)
